# Concurrent mental health and substance use disorders among Canadian adults during the COVID-19 pandemic: a population-based study

**DOI:** 10.24095/hpcdp.46.6.02

**Published:** 2026-06

**Authors:** Cindy Feng, Mark Asbridge

**Affiliations:** Department of Community Health and Epidemiology, Faculty of Medicine, Dalhousie University, Halifax, Nova Scotia, Canada

**Keywords:** concurrent disorders, mental health disorders, substance use disorders, COVID-19 pandemic

## Abstract

**Introduction:**

Concurrent disorders, defined here as co-occurring mental health disorders (MHD) and substance use disorders (SUD), pose challenges for treatment and public health. This study examines the prevalence and characteristics associated with MHD only, SUD only, and concurrent disorders among Canadians aged 15 and older during the COVID-19 pandemic.

**Methods:**

We analyzed data from the 2022 Mental Health and Access to Care Survey (MHACS), a cross-sectional survey of Canadians aged 15 and older living in the 10 provinces (n = 9861). MHD and SUD were assessed using the WHO Composite International Diagnostic Interview. Respondents were classified into four groups: no disorder, MHD only, SUD only, and concurrent disorders. Multinomial logistic regression identified sociodemographic, health, and pandemic-related characteristics associated with these disorder categories, using survey weights and bootstrap methods.

**Results:**

Among respondents, 1.6% had concurrent disorders, 12.2% had a MHD only, and 1.6% had a SUD only. Younger adults, especially those aged 20 to 24, and 2SLGBTQI+ individuals had elevated risk for concurrent disorders. Additional correlates included lower education, rural residence, weak sense of belonging, and functional impairment. Pandemic-related stressors—loneliness, financial hardship, and difficulty accessing care—were strongly associated with concurrent disorders.

**Conclusion:**

This study highlights the prevalence and key correlates for MHD, SUD, and concurrent disorders among Canadian adults during the COVID-19 pandemic. Vulnerable populations include younger individuals, sexual and gender minorities, and those facing social isolation or unmet care needs. These findings underscore the importance of ensuring integrated, accessible mental health and substance use services in Canada’s postpandemic recovery.

## Highlights

• The co-occurrence of mental health and substance use disorders (concurrent disorders) rose from 1.2% in 2012 to 1.6% in 2022.

• The prevalence of mental health disorders alone nearly doubled, from 6.1% in 2012 to 12.2% in 2022, while substance use disorders alone declined from 3.8% to 1.6%.

• Concurrent disorders are most common among young adults (20–24 years), 2SLGBTQI+ individuals, and those with a weak sense of social belonging.

• People in rural areas, those with lower education, and those facing unmet care needs had higher rates of these disorders.

• Pandemic-related stressors, such as loneliness and financial hardship, were strongly linked to the risk of all disorders.

## Introduction

Mental health disorders (MHD) and substance use disorders (SUD) are among the most pressing and interconnected public health challenges worldwide.^1^^,^^2^ These often chronic conditions contribute substantially to disability, reduced productivity, and mortality.^1^^,^^3^ In Canada, nearly one in three people have met the criteria for at least one selected MHD and SUD in their lifetime.^4^

Of particular concern is the frequent co-occurrence of MHD and SUD, often referred to as concurrent disorders or dual diagnoses,^5^ with an estimated past 12-month prevalence of approximately 1.2% based on 2012 Canadian Community Health Survey data.^6^ Concurrent disorders are associated with more severe clinical symptoms, poorer social outcomes, and greater barriers to care.^7^^,^^8^^,^^9^ The concurrent disorders are believed to result from a complex interplay of shared risk factors and bidirectional causal relationships. These include early-life adversities such as trauma and abuse,^10^^,^^11^ chronic stress exposure,^12^ genetic vulnerability,^13^ and overlapping neurobiological pathways.^14^ Individuals with untreated depression or anxiety may turn to substance use as a form of self-medication, while prolonged substance use can in turn exacerbate or precipitate mental illness.^14^^,^^15^^,^^16^^,^^17^^,^^18^ These mechanisms often result in a reinforcing cycle of worsening symptoms, higher rates of emergency care use, and lower treatment retention when care is fragmented.^9^^,^^19^^,^^20^

Despite the well-recognized clinical significance of concurrent disorders, most epidemiologic research has historically examined MHD and SUD as separate conditions, limiting our understanding of their overlap in population settings. Much of the existing literature is based on clinical or treatment-seeking samples,^21^^,^^22^ which may not reflect the full range of disease burden in the general population. While some studies on concurrent disorders exist, many focus on specific subgroups such as adolescents, clinical populations, or individuals who are homeless or marginally housed.^23^^,^^24^^,^^25^ In Canada, most population-level research relies on survey data collected over a decade ago,^6^^,^^8^ which may not reflect current trends or the evolving landscape of concurrent disorders. There is a critical need for up-to-date, population-based studies to better understand the concurrent disorders and to inform effective public health planning.

Crucially, understanding this evolving landscape requires examining how social and structural determinants shaped these co-occurring conditions.^26^^,^^27^^,^^28^ Emerging evidence shows that the COVID-19 pandemic was associated with increased symptoms of depression and anxiety, loneliness and social isolation, financial hardship, and disruptions in access to mental health and addiction services.^29^^,^^30^^,^^31^ These stressors and service disruptions likely amplified pre-existing vulnerabilities and interacted with them, contributing to complex and heterogeneous patterns of MHD and SUD during this period. However, most pandemic-era studies have examined MHD and SUD separately, with relatively little population-based evidence on their co-occurrence.^22^^,^^29^^,^^30^^,^^31^^,^^32^^,^^33^^,^^34^^,^^35^^,^^36^

To address these gaps, we used data from the Mental Health and Access to Care Survey (MHACS),^37^ a population-based survey conducted in 2022 that included Canadians aged 15 and older living in the 10 provinces. Collected in the later phase of the COVID-19 pandemic, these data provide a snapshot of the burden of MHD, SUD, and their co-occurrence during a period of ongoing social disruption and health system strain.

## Methods

### Study design and data source

This study analyzed data from the Public Use Microdata File (PUMF) of MHACS, a cross-sectional survey conducted by Statistics Canada between 17 March and 31 July 2022.^37^ The target population included individuals aged 15 and older living in the 10 Canadian provinces, excluding those living on reserves, full-time members of the Canadian Forces, and residents of collective dwellings (e.g. institutions). The MHACS was designed to assess mental health and access to care in the context of the COVID-19 pandemic, capturing detailed information on psychological symptoms, service use, and sociodemographic characteristics. The MHACS sample was drawn from respondents to the 2021 long-form Census. Of the 39 485 households invited, 9861 completed the survey, yielding a response rate of 25%. To ensure representativeness of the household population in the provinces, Statistics Canada applied sampling weights that account for the survey’s complex design and non-response.

### Study variables and measures

#### Outcome variable

MHDs and SUDs were assessed using the Canadian adaptation of the World Health Organization Composite International Diagnostic Interview (WHO-CIDI),^38^ a standardized and widely validated diagnostic tool designed for epidemiological research. Administered by trained interviewers, the WHO-CIDI applies diagnostic criteria from the *Diagnostic and Statistical Manual of Mental Disorders* (DSM) and the *International Classification of Diseases* (ICD) to identify psychiatric conditions.

This study focused on past-year diagnoses (within 12 months of the survey) to capture the period most affected by the COVID-19 pandemic. MHDs were defined as mood and/or anxiety disorders (e.g. major depressive episode, bipolar I/II, hypomania, generalized anxiety disorder, social phobia). SUDs included alcohol or drug abuse/dependence. Each was coded as present versus not present, and respondents were classified as:

• No disorder: No past-year MHD or SUD

• MHD only: One or more MHD, but no SUD in the past year

• SUD only: One or more SUD, but no MHD in the past year

• Concurrent disorders: At least one MHD and at least one SUD in the past year.

#### Covariates

We included a broad set of covariates to explore individual, social, and contextual factors associated with disorder status, grouped as follows:

### Demographic and socioeconomic characteristics

• Age: 15 to 19, 20 to 24, 25 to 29, 30 to 34, 35 to 44, 45 to 54, 55 to 64, and 65 and older.

• Gender: Men+ and Women+, based on the Statistics Canada-derived gender variable. In the MHACS, records for non-binary respondents are randomly allocated to these two categories for confidentiality; disaggregated non-binary data are not available.

• 2SLGBTQI+ status: Classified as 2SLGBTQI+ vs. non-2SLGBTQI+.

• Marital status: Married or living common-law; never married; separated, divorced or widowed.

• Education level: Less than high school; high school; trades or college or university below bachelor’s; bachelor’s or higher.

• Household income: Low (< $30 000), middle ($30 000–99 999), high (≥ $100 000).

• Place of residence (based on population size): rural (< 1000), small (1000–29 999), medium (30 000–99 999), and large (≥ 100 000).

#### Psychosocial factors

• Sense of social belonging: Very strong, somewhat strong, somewhat weak, very weak.

#### Health status and perceived needs

• Functional impairment: Measured via the World Health Organization Disability Assessment Schedule (WHODAS) 2.0,^39^ which evaluates limitations across six domains (mobility, self-care, social participation, etc.). Scores range from 0 to 40, with higher scores indicating more impairment.

• Perceived need for mental health care in the past year: Respondents were classified into the following four mutually exclusive categories.

– No perceived need: Did not feel a need and did not receive care

– All needs met: Needed care and received it fully

– Partially met needs: Received some care but felt it was insufficient

– Unmet needs: Felt a need but received no care.

#### COVID-19-related stressors

Three binary variables captured pandemic-specific stressors. Respondents were asked whether, due to the COVID-19 pandemic, they had: (1) experienced financial difficulties (financial impact: yes vs. no); (2) had difficulty accessing needed mental health care (access barriers: yes vs. no); and (3) felt lonely or isolated as a result of the pandemic (loneliness: yes vs. no).

### Statistical analysis

Descriptive statistics were used to summarize the sample characteristics overall and by MHD and SUD categories. Continuous variables were presented as medians with interquartile ranges. Categorical variables were summarized using frequencies and weighted percentages.

Multinomial logistic regression was used to compare three diagnostic groups (MHD only, SUD only, concurrent disorders) against a reference group of no disorder. To further differentiate concurrent disorders from single disorders, we refit the models using MHD only and SUD only as alternative reference groups. This strategy distinguishes correlates associated with any disorder (relative to no disorder) from those more specific to concurrent disorders (relative to MHD only or SUD only). Univariate models were fitted for each covariate, followed by a multivariable model including all covariates. Relative risk ratios (RRRs) and adjusted RRRs (aRRRs), along with their 95% confidence intervals (CIs), were reported.

All analyses were conducted in Stata version 18 (StataCorp LLC, College Station, TX, USA). Person-level survey weights were applied to obtain population-representative estimates. Variance estimation and 95% CIs used the bootstrap replicate weights provided by the MHACS, implemented with Stata’s survey procedures for bootstrap replicate designs, which account for the complex sampling design.

### Ethics approval

This study utilized publicly available, de-identified secondary data from the MHACS. According to the Tri-Council Policy Statement (TCPS 2), research using anonymized, publicly accessible secondary data is exempt from institutional ethics review.

## Results

Table 1 summarizes participant characteristics by diagnostic category. Most respondents (84.6%) reported no past-year MHD or SUD, while 12.2% had MHD only, 1.6% had SUD only, and 1.6% had concurrent disorders. Concurrent disorders were most prevalent among adults aged 20 to 24 (6.2%) and 2SLGBTQI+ respondents (8.3%), and were more common among individuals who were never married, those with lower educational attainment, and those living in rural areas. Higher prevalence of concurrent disorders was also observed among participants reporting weaker social belonging, chronic physical conditions, greater functional impairment, and unmet mental health care needs. Pandemic-related stressors, including loneliness, financial strain, and access barriers, were reported more often among individuals with concurrent disorders.

**Table 1:** Participant characteristics stratified by diagnostic category, MHACS, 2022

**Variable**	**Category**	**No disorder** (n = 7236) n (%)	**MHD only** (n = 1043) n (%)	**SUD only** (n = 133) n (%)	**Concurrent** (n = 137) n (%)	**Total** (n = 8549) n (%)
**Demographic and socioeconomic characteristics**						
**Age group** (n = 8549)	15–19	584 (70.0%)	188 (24.3%)	12 (2.0%)	18 (3.8%)	802
	20–24	687 (66.9%)	242 (22.3%)	38 (4.7%)	51 (6.2%)	1 018
	25–29	362 (73.0%)	105 (20.9%)	8 (1.7%)	18 (4.4%)	493
	30–34	472 (76.3%)	83 (16.3%)	14 (4.1%)	13 (3.3%)	582
	35–44	1 042 (81.9%)	134 (14.2%)	18 (1.9%)	18 (2.1%)	1 212
	45–54	926 (84.5%)	111 (12.4%)	15 (2.0%)	9 (1.1%)	1 061
	55–64	1 028 (89.7%)	95 (9.0%)	8 (0.7%)	6 (0.6%)	1 137
	65+	2 135 (94.4%)	85 (4.5%)	20 (0.9%)	4 (0.2%)	2 244
**Gender** (n = 8536)	Women+	3 449 (79.9%)	690 (16.9%)	36 (1.1%)	66 (2.1%)	4 241
	Men+	3 776 (86.4%)	351 (9.0%)	97 (2.7%)	71 (2.0%)	4 295
**2SLGBTQI+** (n = 8307)	No	6 731 (85.0%)	844 (11.6%)	118 (1.8%)	96 (1.6%)	7 789
	Yes	297 (56.1%)	170 (33.2%)	12 (2.4%)	39 (8.3%)	518
**Marital status** (n = 8508)	Married or living common law	4 248 (88.4%)	345 (9.6%)	48 (1.2%)	27 (0.8%)	4 668
	Never married	2 054 (72.4%)	594 (20.3%)	72 (3.1%)	95 (4.2%)	2 815
	Separated, divorced or widowed	904 (85.1%)	97 (11.1%)	11 (1.8%)	13 (2.0%)	1 025
**Education** (n = 8360)	Less than high school	617 (78.7%)	125 (15.9%)	10 (2.1%)	15 (3.3%)	767
	High school	1 545 (79.2%)	310 (15.5%)	42 (2.5%)	55 (2.8%)	1 952
	Trade, college or university below bachelor	2 132 (83.7%)	281 (12.4%)	45 (1.9%)	40 (1.9%)	2 498
	Bachelor’s degree or higher	2 776 (86.9%)	310 (10.9%)	32 (1.2%)	25 (1.0%)	3 143
**Household income** (n = 8497)	Low income	467 (79.8%)	71 (15.9%)	10 (1.8%)	9 (2.5%)	557
	Middle income	2 804 (83.8%)	348 (11.8%)	47 (1.9%)	57 (2.5%)	3 256
	High income	3 922 (82.9%)	615 (13.6%)	76 (1.9%)	71 (1.6%)	4 684
**Place of residence** (n = 8549)	Rural (< 1000)	954 (84.0%)	121 (11.2%)	26 (2.5%)	20 (2.3%)	1 121
	Small (1000–29 999)	627 (80.3%)	101 (15.1%)	12 (1.9%)	17 (2.6%)	757
	Medium (30 000–99 999)	517 (83.9%)	77 (13.1%)	9 (1.6%)	9 (1.4%)	612
	Large (≥ 100 000)	5 138 (83.2%)	744 (13.1%)	86 (1.7%)	91 (1.9%)	6 059
**Psychosocial factors**						
**Sense of belonging** (n = 8362)	Very strong	1 326 (92.7%)	73 (5.5%)	13 (1.5%)	4 (0.4%)	1 416
	Somewhat strong	3 554 (87.2%)	387 (9.7%)	63 (1.8%)	45 (1.2%)	4 049
	Somewhat weak	1 720 (75.7%)	393 (19.0%)	40 (1.9%)	58 (3.4%)	2 211
	Very weak	473 (65.9%)	168 (26.0%)	16 (3.0%)	29 (5.1%)	686
**Health status and perceived needs**						
**Functional impairment** (n = 8217)	WHO disability score	0 (6)	11 (19)	3 (11)	14 (19)	3 (8)
**Perceived needs** (n = 8301)	No perceived need for mental health	5 998 (93.2%)	305 (4.8%)	78 (1.4%)	27 (0.5%)	6 408
	All needs met	674 (63.5%)	308 (28.9%)	27 (3.0%)	42 (4.6%)	1 051
	Partially met	189 (38.5%)	249 (50.4%)	16 (3.3%)	34 (7.9%)	488
	Not met	202 (56.5%)	126 (34.4%)	7 (2.8%)	19 (6.3%)	354
**COVID-19-related stressors**						
**Financial impact** (n = 8487)	No	5 751 (85.9%)	706 (11.2%)	81 (1.5%)	69 (1.4%)	6 607
	Yes	1 434 (74.0%)	329 (18.8%)	49 (3.0%)	68 (4.1%)	1 880
**Access barriers** (n = 8487)	No	6 017 (85.3%)	744 (11.2%)	106 (2.0%)	80 (1.5%)	6 947
	Yes	1 168 (74.2%)	291 (20.3%)	24 (1.4%)	57 (4.1%)	1 540
**Loneliness** (n = 8487)	No	4 177 (92.8%)	215 (5.4%)	40 (1.2%)	18 (0.6%)	4 450
	Yes	3 008 (73.0%)	820 (20.9%)	90 (2.5%)	119 (3.5%)	4 037

**Abbreviations:** MHACS, Mental Health and Access to Care Survey; MHD, mental health disorders; pop, population; SUD, substance use disorders; WHO, World Health Organization.

**Notes:** Values are presented as counts (percentages) unless otherwise noted.

WHO disability score (WHODAS 2.0)^38^ is presented as median (interquartile range).

Table S1 (supplementary materials) presents unadjusted RRRs from univariate multinomial regression. Younger age, 2SLGBTQI+ identity, never married status, lower income and education, rural residence, weaker social belonging, greater disability, unmet mental health care needs, and pandemic-related stressors were all associated with higher risk of concurrent disorders.

**Figure 1:**
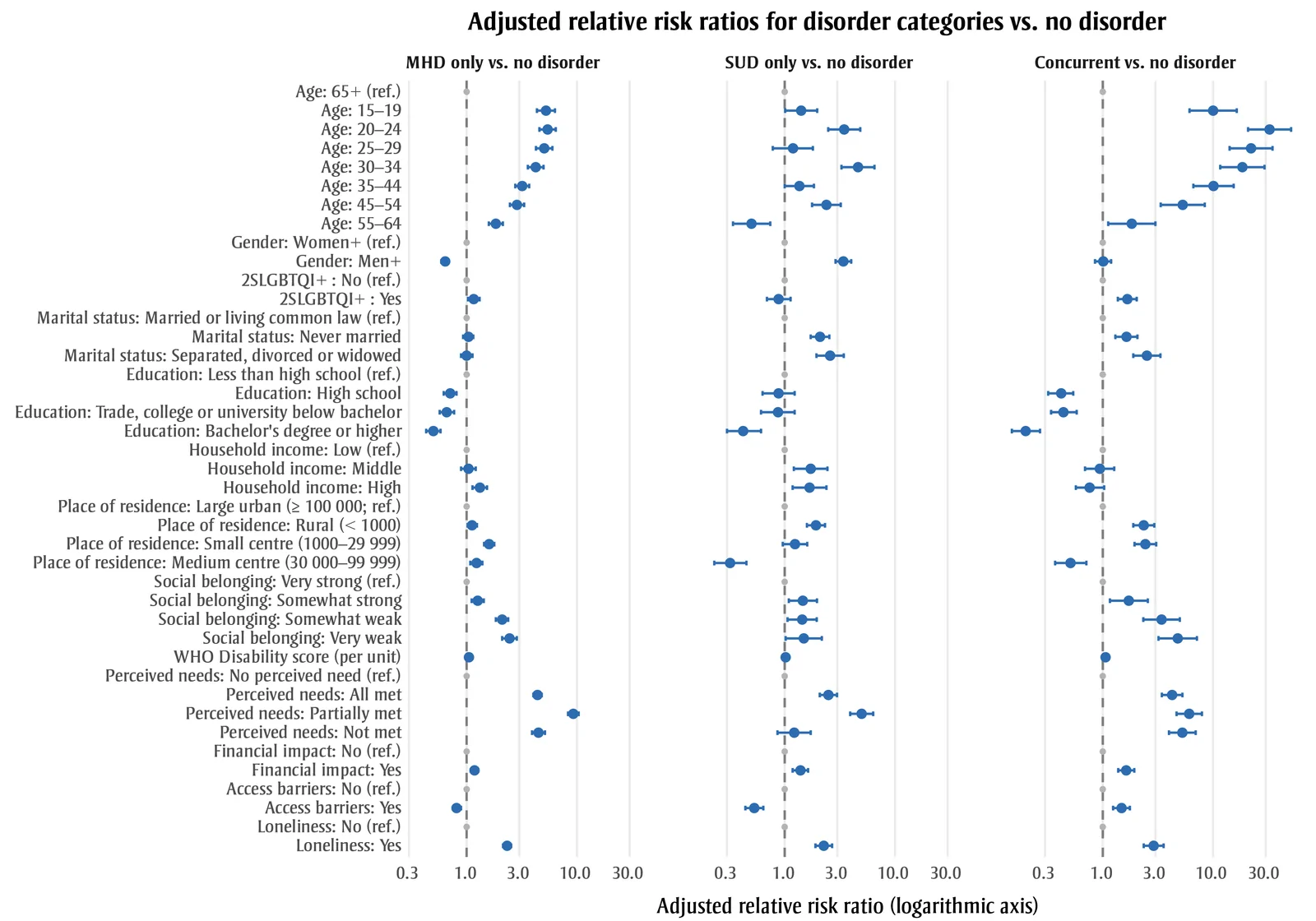
Adjusted relative risk ratios (aRRRs) and 95% CIs from multivariable multinomial logistic regression models comparing MHD only, SUD only, and concurrent disorders to the reference category of no disorder (MHACS, 2022; n = 7447) **Abbreviations:** CIs, confidence intervals; MHACS, Mental Health and Access to Care Survey; MHD, mental health disorders; ref., referent; SUD, substance use disorders; WHO, World Health Organization.

Table 2 presents aRRRs from the multivariable multinomial models. Figure 1 displays aRRRs for each disorder category using “no disorder” as the reference group, and Figure S1 (supplementary materials) presents aRRRs for models using “MHD only” and “SUD only” as alternative reference groups. Younger age was consistently associated with a higher risk of all disorder categories relative to older adults (65+). The risk of concurrent disorders was particularly elevated among individuals aged 20 to 24 (aRRR = 32.48; 95% CI: 20.73–50.91). Compared to those with MHD only, this age group had nearly six times higher risk of concurrent disorders (aRRR = 5.99). Compared to those with SUD only, the risk of concurrent disorders was also substantially higher among young adults, especially those aged 25 to 29 (aRRR = 16.99) and 20 to 24 (aRRR = 9.38), highlighting early adulthood as a critical period for co-occurrence.

**Table 2:** Adjusted relative risk ratios (aRRRs) from multivariable multinomial logistic regression models assessing associations between covariates and three disorder categories: MHD only, SUD only, and concurrent disorders, MHACS, 2022

**Variable**	**Category**	**MHD only vs.** **no disorder**	**SUD only vs.** **no disorder**	**Concurrent vs.** **no disorder**	**Concurrent vs. MHD only**	**Concurrent vs. SUD only**
		**aRRR (95% CI)**	**aRRR (95% CI)**	**aRRR (95% CI)**	**aRRR (95% CI)**	**aRRR (95% CI)**
**Demographic and socioeconomic characteristics**						
**Age** (ref.: 65+)	15–19	5.24 (4.34–6.32)***	1.41 (1.01–1.97)*	10.00 (6.11–16.37)***	1.91 (1.15–3.17)**	7.11 (3.96–12.77)***
	20–24	5.42 (4.57–6.43)***	3.46 (2.48–4.84)***	32.48 (20.73–50.91)***	5.99 (3.78–9.49)***	9.38 (5.42–16.23)***
	25–29	5.05 (4.25–5.99)***	1.19 (0.78–1.80)	22.08 (14.11–34.55)***	3.99 (2.51–6.34)***	16.99 (9.31–31.00)***
	30–34	4.23 (3.59–4.98)***	4.62 (3.27–6.51)***	18.42 (11.60–29.23)***	4.36 (2.72–6.99)***	3.99 (2.27–7.00)***
	35–44	3.19 (2.76–3.69)***	1.36 (1.00–1.84)*	10.08 (6.62–15.35)***	3.16 (2.05–4.87)***	7.42 (4.47–12.31)***
	45–54	2.86 (2.47–3.32)***	2.39 (1.77–3.22)***	5.30 (3.35–8.40)***	1.85 (1.16–2.97)**	2.22 (1.29–3.84)***
	55–64	1.84 (1.59–2.13)***	0.50 (0.34–0.74)***	1.83 (1.12–3.00)*	1.00 (0.60–1.65)	3.63 (1.98–6.66)***
**Gender** (ref.: Women+)	Men+	0.64 (0.59–0.68)***	3.40 (2.89–4.01)***	1.01 (0.85–1.19)	1.59 (1.34–1.88)***	0.30 (0.24–0.37)***
**2SLGBTQI+** (ref.: No)	Yes	1.16 (1.03–1.31)*	0.88 (0.69–1.13)	1.67 (1.37–2.03)***	1.44 (1.19–1.74)***	1.89 (1.40–2.55)***
**Marital status** (ref.: Married or living common law)	Never married	1.04 (0.93–1.15)	2.09 (1.72–2.54)***	1.64 (1.30–2.06)***	1.58 (1.26–1.99)***	0.78 (0.58–1.05)
	Separated, divorced or widowed	1.00 (0.89–1.13)	2.58 (1.94–3.43)***	2.51 (1.89–3.33)***	2.50 (1.89–3.31)***	0.97 (0.66–1.44)
**Education** (ref.: Less than high school)	High school	0.71 (0.62–0.81)***	0.88 (0.63–1.23)	0.42 (0.32–0.54)***	0.59 (0.45–0.77)***	0.47 (0.32–0.71)***
	Trade, college, or university below bachelor	0.66 (0.57–0.77)***	0.87 (0.61–1.23)	0.44 (0.34–0.58)***	0.67 (0.50–0.90)**	0.51 (0.33–0.78)***
	Bachelor’s degree or higher	0.50 (0.43–0.58)***	0.42 (0.30–0.61)***	0.20 (0.15–0.27)***	0.40 (0.30–0.55)***	0.47 (0.30–0.74)***
**Household income** (ref.: Low)	Middle	1.04 (0.89–1.21)	1.72 (1.21–2.44)**	0.94 (0.69–1.27)	0.90 (0.66–1.23)	0.55 (0.35–0.86)**
	High	1.32 (1.13–1.53)***	1.68 (1.18–2.39)**	0.76 (0.57–1.03)	0.58 (0.42–0.79)***	0.45 (0.29–0.72)***
**Place of residence** (ref.: Large [≥ 100 000])	Rural (< 1000)	1.12 (1.02–1.24)*	1.92 (1.59–2.32)***	2.35 (1.89–2.93)***	2.09 (1.68–2.61)***	1.22 (0.92–1.63)
	Small (1000–29 999)	1.60 (1.44–1.78)***	1.24 (0.96–1.60)	2.43 (1.94–3.05)***	1.52 (1.21–1.91)***	1.96 (1.41–2.74)***
	Medium (30 000–99 999)	1.23 (1.08–1.39)**	0.32 (0.23–0.45)***	0.51 (0.37–0.71)***	0.42 (0.30–0.58)***	1.60 (1.01–2.54)**
**Psychosocial factors**						
**Social belonging** (ref.: Very strong)	Somewhat strong	1.26 (1.11–1.43)***	1.46 (1.09–1.96)*	1.72 (1.16–2.56)**	1.37 (0.92–2.04)	1.18 (0.72–1.92)
	Somewhat weak	2.10 (1.84–2.38)***	1.44 (1.06–1.95)*	3.41 (2.33–4.99)***	1.63 (1.11–2.38)**	2.37 (1.46–3.87)***
	Very weak	2.45 (2.10–2.85)***	1.49 (1.02–2.17)*	4.78 (3.20–7.13)***	1.95 (1.32–2.89)***	3.20 (1.88–5.46)***
**Health status and perceived needs**						
**Functional impairment**	WHO disability score	1.05 (1.05–1.06)***	1.02 (1.01–1.03)***	1.06 (1.06–1.07)***	1.01 (1.00–1.02)**	1.04 (1.03–1.05)***
**Perceived needs** (ref.: No perceived need)	All met	4.39 (4.03–4.79)***	2.49 (2.08–2.98)***	4.25 (3.43–5.27)***	0.97 (0.77–1.21)	1.71 (1.30–2.24)***
	Partially met	9.27 (8.30–10.36)***	4.98 (3.92–6.34)***	6.07 (4.66–7.92)***	0.66 (0.50–0.86)***	1.22 (0.87–1.70)
	Not met	4.48 (3.92–5.13)***	1.22 (0.86–1.72)	5.26 (3.98–6.95)***	1.17 (0.88–1.56)	4.32 (2.82–6.61)***
**COVID-19-related stressors**						
**Financial impact** (ref.: No)	Yes	1.18 (1.09–1.27)***	1.39 (1.18–1.63)***	1.63 (1.38–1.93)***	1.38 (1.17–1.64)***	1.18 (0.94–1.48)
**Access barriers** (ref.: No)	Yes	0.81 (0.75–0.89)***	0.53 (0.44–0.64)***	1.48 (1.24–1.76)***	1.81 (1.52–2.16)***	2.77 (2.17–3.52)***
**Loneliness** (ref.: No)	Yes	2.33 (2.14–2.53)***	2.26 (1.90–2.69)***	2.89 (2.35–3.56)***	1.24 (0.99–1.56)	1.28 (0.98–1.67)

**Abbreviations:** aRRR, adjusted relative risk ratio; CIs, confidence intervals; MHACS, Mental Health and Access to Care Survey; MHD, mental health disorders; pop, population; ref., referent; SUD, substance use disorders; WHO, World Health Organization.

**Note:** Models used different reference groups—no disorder, MHD only, and SUD only—to specifically compare concurrent disorders against each category (MHACS sample: n = 7447).

* *p* < 0.05.

** *p* < 0.01.

*** *p* < 0.001.

Men were less likely to report MHD only (aRRR = 0.64) but more likely to report SUD only (aRRR = 3.40) versus no disorder. Compared with MHD only, men had higher risk of concurrent disorders (aRRR = 1.59), but lower risk compared to SUD only (aRRR = 0.30), suggesting SUD often occurs alone among men. 2SLGBTQI+ individuals had greater risk of concurrent disorders relative to those with no disorder (aRRR = 1.67) compared with non-2SLGBTQI+ individuals, and also had elevated risk of concurrent disorders relative to MHD only (aRRR = 1.44) and SUD only (aRRR = 1.89). Compared with those who were married or living common-law, individuals who had never been married or were separated, divorced or widowed were at increased risk of SUD only and concurrent disorders relative to no disorder. These groups also showed elevated risk of concurrent disorders relative to MHD only (aRRRs = 1.58 and 2.50, respectively), though not significantly different from SUD only.

Higher education was a strong protective factor: respondents with a bachelor’s degree or higher had reduced risks of all disorder types and were less likely to report concurrent disorders relative to MHD only (aRRR = 0.40) or SUD only (aRRR = 0.47). Middle and high income were associated with higher risk of SUD only compared to no disorder, but high income was inversely associated with concurrent disorders relative to both MHD only (aRRR = 0.58) and SUD only (aRRR = 0.45).

Place of residence showed complex associations. Compared to residents of large urban centres, rural residents had increased risk of SUD only (aRRR = 1.92) and concurrent disorders (aRRR = 2.35) versus no disorder and were more likely to report concurrent disorders versus MHD only (aRRR = 2.09). Residents of small population centres also had elevated risk of MHD only and concurrent disorders relative to no disorder (aRRRs = 1.60 and 2.43, respectively), and were more likely to report concurrent disorders relative to both MHD only (aRRR = 1.52) and SUD only (aRRR = 1.96). Residents of medium-sized centres were significantly more likely to have concurrent disorders compared to those with SUD only (aRRR = 1.60), despite overall reduced risk for SUD.

A clear gradient was observed with social belonging: compared with those reporting very strong belonging, individuals with very weak belonging had substantially higher risk of concurrent disorders versus no disorder (aRRR = 4.78) and versus MHD only (aRRR = 1.95) or SUD only (aRRR = 3.20). Higher disability scores were also associated with increased risk of all disorder categories, with particularly elevated risk for concurrent disorders (aRRR = 1.06). Disability remained positively associated with concurrent disorders relative to both MHD only (aRRR = 1.01) and SUD only (aRRR = 1.04). Perceived need for mental health care was strongly associated with disorder status: relative to no perceived need, respondents with all needs met, partially met, or unmet needs had higher risk of concurrent disorders (aRRR = 4.25, 6.07, and 5.26, respectively). In comparisons between diagnostic groups, partially met needs were associated with a lower risk of concurrent versus MHD only (aRRR = 0.66), whereas unmet needs were associated with a higher risk of concurrent versus SUD only (aRRR = 4.32).

Pandemic-related stressors were significantly associated with all outcomes. Financial difficulties due to COVID-19 were linked to higher risk of MHD only, SUD only, and concurrent disorders versus no disorder (aRRR = 1.18, 1.39, and 1.63, respectively), and to higher risk of concurrent disorders versus MHD only (aRRR = 1.38). Respondents reporting access barriers to mental health care services had lower risk of MHD only and SUD only versus no disorder (aRRR = 0.81 and 0.53) but higher risk of concurrent disorders versus no disorder (aRRR = 1.48) and versus MHD only and SUD only (aRRR = 1.81 and 2.77). Loneliness was associated with higher risk of all disorder categories versus no disorder, with the strongest association for concurrent disorders (aRRR = 2.89), although differences between concurrent and single-disorder groups were not statistically significant.

## Discussion

This study provides one of the first population-based examinations of concurrent disorders among Canadian adults during the COVID-19 pandemic, using data from MHACS, which covers the 10 provinces. We found that approximately 1.6% of adults experienced concurrent disorders, 12.2% had MHD only, and 1.6% had SUD only. To contextualize these findings, we compared them with the most recent pre-pandemic national data from the 2012 Canadian Community Health Survey-Mental Health (CCHS-MH),^6^ which remains the latest population-based pre-pandemic survey using WHO-CIDI diagnostic measures. The prevalence of concurrent disorders rose modestly from 1.2% to 1.6%, MHD only nearly doubled (6.1% to 12.2%), while SUD only declined (3.8% to 1.6%).

Taken together, these patterns suggest a shift toward a higher burden of MHD and a smaller proportion of individuals with isolated SUD. This change likely reflects the cumulative influence of multiple factors over the past decade (e.g. greater awareness, evolving diagnostic and help-seeking practices, and broader societal stressors), with the COVID-19 pandemic as an important, but not sole contributor. Comparisons between 2012 and 2022 should also be made cautiously: the MHACS (2022) used telephone interviews, whereas CCHS-MH (2012) relied primarily on in-person interviews, and other methodological differences may affect estimated prevalence. As a result, the MHACS is best viewed as a post-2020 snapshot that can be compared with 2012 estimates but not used to determine why prevalence changed over time.

Our results highlight that younger age was strongly associated with all disorder categories. Young adults had the highest risks of concurrent disorders; for example, those aged 20 to 24 had over 30 times the risk compared with adults aged 65 and older. This pattern likely reflects both the higher prevalence of MHD and SUD individually in late adolescence and early adulthood, and the social and economic transitions of this period (e.g. schooling, employment, relationships) that can increase vulnerability to both.^12^^,^^40^ However, given the cross-sectional design, we cannot determine whether early adulthood represents a distinct etiologic “high-risk window” for comorbidity or primarily reflects age-related patterns in mental health and substance use that tend to attenuate across the life course.

Gender patterns showed expected trends: men had lower risk of MHD only (aRRR = 0.64), higher risk of SUD only (aRRR = 3.40), and higher risk of concurrent disorders compared to MHD only (aRRR = 1.59). However, men were far less likely to have concurrent disorders compared to SUD only (aRRR = 0.30), suggesting substance use among men may more often occur in isolation rather than in conjunction with mental health issues.^6^^,^^14^^,^^26^

2SLGBTQI+ individuals had a 67% higher risk of concurrent disorders versus no disorder compared to non-2SLGBTQI+ respondents and were more likely to experience comorbidity than either disorder alone, consistent with literature documenting heightened pandemic-related disparities in sexual and gender minorities.^32^^,^^33^^,^^41^ These differences likely stem from stigma, discrimination, and barriers to accessing affirming care, underscoring the need for inclusive and culturally competent mental health and addiction service.^32^^,^^33^^,^^41^

Marital status was significantly associated with disorder categories: individuals who had never been married and those who were separated, divorced, or widowed had higher relative risks of SUD and concurrent disorders compared to those who were married or living common-law. This aligns with existing literature suggesting that marriage may be linked to greater emotional support and social integration, which could buffer stress.^42^^,^^43^^,^^44^ However, our models did not include an interaction between marital status and gender, so these estimates reflect average associations across genders and may not capture potential gender differences.

Educational attainment showed a consistent inverse association with all disorder types, with individuals holding a bachelor’s degree or higher exhibiting substantially lower relative risks. This is consistent with research indicating that higher education correlates with enhanced socioeconomic stability, health literacy, and access to protective resources.^45^

Income effects were nuanced, revealing a clear distinction between drivers of severe comorbidity and single SUD. Middle and high incomes were associated with a decreased risk of concurrent disorders, supporting the view that greater economic resources act as a protective buffer against the chronic stress and instability that precipitate the most severe dual diagnoses.^8^^,^^20^ Conversely, the same income levels were associated with an increased risk of SUD only. This divergence suggests substance use in affluent groups is often driven by social normalization or recreational factors,^46^^,^^47^ independent of the overwhelming psychological distress associated with concurrent disorders.

Our findings reveal notable geographic disparities in the prevalence of MHD, SUD, and their co-occurrence. Individuals living in rural and small population centres exhibited higher risks of MHD only and especially of SUD only and concurrent disorders compared to those in large urban areas. In contrast, residents of medium-sized centres had elevated MHD risk but significantly lower risks for SUD and concurrent disorders. These patterns may reflect differences in access to healthcare services, social supports, and environmental stressors outside major urban centres,^26^^,^^48^ underscoring the need for tailored public health strategies that address the unique challenges faced by rural and smaller communities.

Social belonging showed a clear gradient: weaker belonging was associated with substantially higher risks of all disorder categories, with very weak belonging linked to nearly fivefold higher risk of comorbidity. This association may reflect both the social consequences of living with MHD or SUD and the possibility that low perceived belonging increases vulnerability to these conditions. Given the cross-sectional design, we cannot infer directionality, but the gradient suggests that social integration and community connectedness are important considerations for prevention and treatment planning.

The strong association between functional impairment and concurrent disorders is consistent with prior work showing that comorbid problems are linked to greater disability and role disruption than single disorders alone.^49^^,^^50^ In our study, higher WHODAS 2.0 scores were associated with an increased relative risk of all disorder categories, with the largest effect observed for concurrent disorders. This pattern may reflect both the added functional burden of managing co-occurring conditions and the possibility that greater disability increases vulnerability to developing comorbid MHD and SUD, for example through reduced employment, social participation, or access to care. Given the cross-sectional design, we cannot disentangle these pathways, but the findings underscore the importance of integrating functional assessment and rehabilitation supports within services for people with concurrent disorders.

Finally, COVID-19-related financial hardship, loneliness, and disrupted access to care were strongly associated with all disorder categories, particularly concurrent disorders.^34^^,^^35^^,^^36^ Loneliness nearly tripled risk of concurrent disorders, while access barriers increased comorbidity risk by over 80% compared to MHD only and nearly 180% compared to SUD only. These relationships are likely bidirectional: people with MHD and SUD may be more vulnerable to job loss, isolation, and difficulties navigating care, while these stressors may also worsen or precipitate disorders. Because MHACS is cross-sectional, we cannot determine temporal ordering, but the strong associations highlight the need to address both psychosocial and structural factors in post-pandemic planning.

### Strengths and limitations

This study offers several important contributions. First, its 2022 MHACS data from a population-based sample of Canadians aged 15 and older in the 10 provinces, allowing examination of how pandemic-related stressors (e.g. social isolation, financial hardship, care disruptions) relate to MHD and SUD, and their co-occurrence. Second, multinomial regression enables simultaneous comparison of MHD only, SUD only, and concurrent disorders, providing a more nuanced view than binary models. Third, incorporating diverse social determinants and pandemic-related indicators supports a comprehensive understanding of multiple vulnerabilities to inform public health responses.

Limitations include reliance on self-reported, structured diagnostic interviews rather than clinical assessments, which may result in misclassification. Furthermore, the low survey response rate (25%) introduces a risk of non-response bias, meaning the findings may not accurately represent characteristics of the full target population. The cross-sectional design limits causal inference, and findings may not generalize to non-pandemic settings. Survival bias is also possible, particularly among older respondents, if those with earlier-onset MHD or SUD were more likely to die before the survey. Finally, exclusion of residents in the territories and Indigenous communities living on reserves limits generalizability to these populations.

## Conclusion

This study provides population-based estimates of MHD, SUD, and concurrent disorders among Canadian adults during the COVID-19 pandemic and identifies key sociodemographic, psychosocial, and pandemic-related characteristics associated with these outcomes. Concurrent disorders affected 1.6% of adults and were most prevalent among younger, 2SLGBTQI+, lower socioeconomic, and rural individuals. High-risk factors consistently included COVID-19 stressors: loneliness, financial hardship, and self-reported difficulty accessing needed care. Given the cross-sectional design, we cannot infer causality; however, the strong association highlights a vulnerable population facing substantial barriers to care. These findings suggest that high-risk groups (e.g. younger adults, people experiencing social isolation) may particularly benefit from targeted interventions and from efforts to improve the accessibility and coordination of mental health and substance use services in the postpandemic context.

## Data Availability

None.
